# The Potential Regulatory Roles of Circular RNAs in Tumor Immunology and Immunotherapy

**DOI:** 10.3389/fimmu.2020.617583

**Published:** 2021-02-03

**Authors:** Zhixiao Fang, Chunjie Jiang, Shengli Li

**Affiliations:** ^1^ Institute of Translational Medicine, Shanghai General Hospital, Shanghai Jiao Tong University School of Medicine, Shanghai, China; ^2^ Institute for Diabetes, Obesity, and Metabolism, Perelman School of Medicine at the University of Pennsylvania, Philadelphia, PA, United States; ^3^ Division of Endocrinology, Diabetes, and Metabolism, Department of Medicine, Perelman School of Medicine at the University of Pennsylvania, Philadelphia, PA, United States

**Keywords:** circular RNA, regulatory roles, tumor immunology, immunotherapy, immune checkpoint

## Abstract

Circular RNAs (circRNAs) are covalently closed RNA molecules in eukaryotes with features of high stability, tissue-specific and cell-specific expression. According to their biogenesis, circRNAs are mainly classified into five types, i.e. exonic circRNAs (EciRNAs), exon-intron circRNAs (EIciRNAs), intronic RNAs (CiRNAs), fusion circRNAs (f-circRNAs), and read-through circRNAs (rt-circRNAs). CircRNAs have been emerging as important non-coding regulatory RNAs in a variety of human cancers. CircRNA4s were revealed to exert regulatory function through multiple mechanisms, such as sponges/decoys of miRNAs and proteins, enhancers of protein functions, protein scaffolds, protein recruitment, or protein translation templates. Furthermore, some circRNAs are intensively associated with immune cells in tumor immune microenvironment (TIME), e.g. circARSP91 and natural killer cells. Through regulating immune checkpoint genes, circRNAs are demonstrated to modulate the immune checkpoint blockade immunotherapy, e.g. circCPA4 could up-regulate PD-L1 expression. In summary, we reviewed the molecular features of circRNAs and mechanisms how they exert functions. We further summarized functional implications of circRNA regulations in tumor immunology and immunotherapy. Further understanding of the regulatory roles of circRNAs in tumor immunology and immunotherapy will benefit tumor treatment.

## Introduction

Circular RNAs (circRNAs) are single-stranded circularized RNA molecules produced from back-splicing. Accumulating evidence has shown that circRNA dysregulations are involved in a variety of human disorders, including viral infection (1), cardiac fibrosis (2), diabetes (3), and cancer (4). Advances in high-throughput sequencing technologies and computational algorithms have driven the systematic detection and investigation of circRNAs. Through diverse mechanisms, circRNAs have shown important roles in tumor immunology and immunotherapy. In this review, we summarized the molecular characteristics of circRNAs and how they exert functions through various mechanisms. We further reviewed and discussed the prospective of circRNAs utilities in tumor immunology and immunotherapy.

## The Regulatory Roles of circRNAs in Human Cancers

### Molecular Properties of circRNAs

Circular RNAs (circRNAs) are single-stranded covalently closed RNA molecules, which are generated by “back-splicing” where the spliceosome joins the 3’ end of an exon with an upstream 5’ end of the same or different exons (5). Briefly, the length of circRNAs can be ranging from hundreds of nucleotides to more than 1,000 nucleotides dependent on their host genes. They are highly stable in general due to their covalently closed ring structure. While the turnover of circRNAs are still in investigation, one report indicated that circRNAs with N6-methyladenosine (m6A) modification could be cleaved by the ribonuclease P (RNase P) multi-drug resistance-associated protein 1 (MRP) complex in a way that dependent on YTH domain-containing family protein 2 (YTHDF2) and heart-responsive protein 12 (HRSP12) (6). In another report, CDR1AS could be cleaved by protein argonaute 2 (AGO2) which plays an important role in RNA interference (7). It was initially considered as byproducts generated from aberrant splicing events (8–11). In recent years, the rapid development of high-throughput RNA sequencing (RNA-seq) and bioinformatics methods has promoted the extensive identification of circRNAs in eukaryotes (12–16). CircRNAs are characterized by high stability, widespread expression in diverse species, and high specificity among different species.

#### CircRNA Classification

Most circRNAs are generated from protein-coding genes, which are processed in the exon skipping during pre-messenger RNA (pre-mRNA) transcription to form a lariat structure containing single or multiple exons. This is called exonic circRNAs (EciRNAs) (17–19). Some circRNAs contain both exonic and intronic sequences that are derived from internal intron retention, which are called exon-intron circRNAs (EIciRNAs) (20). Circular intronic RNAs (CiRNAs) are generated from intronic lariats that are kept during canonical splicing process (21). In addition, circRNAs can also be produced from exon joint of different genes located in different or the same chromosomes, which are called fusion circRNAs (f-circRNAs) (22) and read-through circRNAs (rt-circRNAs) (4), respectively.

#### CircRNA Biogenesis

CircRNAs are generated from canonical splice sites from back-splicing, which is partly dependent on the canonical splicing machinery (14, 23) and have been shown to compete with linear RNAs (24). The biogenesis of most circRNAs is affected by cis-acting elements and trans-acting factors (17). In general, circRNAs are produced from looping intron sequences flanking the downstream splice-donor site and upstream splice-acceptor site. This process could be mediated by base pairing between inverted repeat elements or RNA-binding proteins (RBPs), such as QKI and FUS (25, 26). Additionally, during the process of exon skipping, some of excised lariats could undergo internal back-splicing, which would lead to circRNA formation (27). CiRNAs could also be produced from intronic lariats that escape from debranching (21). Other factors influencing circRNA biogenesis include epigenetic changes, such as histone modification and DNA methylation status variations within gene bodies (28, 29).

### The Functional Mechanisms of circRNA Regulation in Human Cancers

Accumulating studies have shown that perturbations of circRNAs is prevalent in human cancers (4, 30), including thyroid cancer (31), ovarian cancer (32), and gastrointestinal cancers (33). Recently, a global analysis of circRNA landscape using clinical tumor samples (>2,000) was performed across more than 40 cancer types (4). Notably, this study identified over 160,000 circRNAs that showed expression in at least one cancer type. Another recent study, specifically in localized prostate cancer, identified 76,311 circRNAs through analyzing RNA-seq data derived from prostate tumor specimens (34). Furthermore, they also found a variety of circRNAs were functionally dysregulated in cancer. In particular, Chen et al. identified 171 circRNAs that were essential to prostate cancer cell proliferation. Collectively, these studies demonstrated the high prevalence of circRNA expression and their perturbations in cancers.

**Figure 1:**
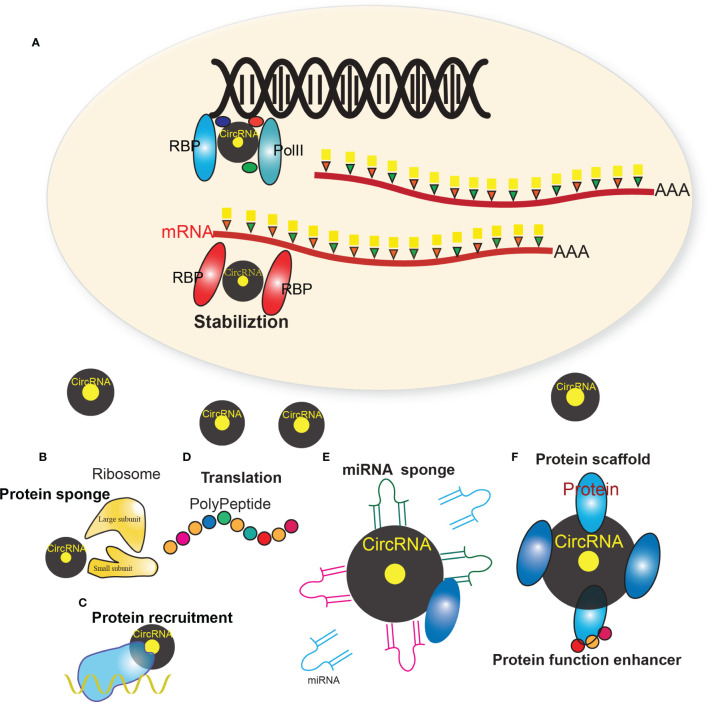
Multiple regulatory mechanism of circRNAs. **(A)** The biogenesis of circRNAs mainly involves the complex of PolII, RBPs and other factors. **(B)** CircRNAs that harbor RNA binding protein (RBP) binding motifs may serve as sponges/decoys of the corresponding proteins and regulate their functions. **(C)** CircRNAs may also recruit specific proteins to certain loci or subcellular compartments. **(D)** Some circRNAs harbor internal ribosome entry site elements and AUG sites, which enables circRNAs to be translated to unique peptides under certain circumstances. **(E)** Many circRNAs were found to act as miRNAs sponges, which sequester miRNAs via complementary RNA base-pairing and thus prevent miRNAs from binding their target. **(F)** Some circRNAs have been shown to facilitate the colocalization of enzymes and their substrates through acting as protein scaffolds. CircRNAs may also enhance the function of particular proteins through circRNA-protein interactions.

CircRNAs can exert their regulatory roles in cancer *via* different ways (17, 35) (**Figure 1**), i.e. protein sponges/decoys (**Figure 1B**) (23), protein recruitment (**Figure 1C**) (36), and templates for translation (**Figure 1D**) (37), miRNA sponges/decoys (**Figure 1E**) (38), protein scaffolding (39), and enhancer of protein function (**Figure 1F**) (20).

#### CircRNAs as miRNA Sponges

Many circRNAs were found to act as miRNAs sponges, which sequester miRNAs *via* complementary RNA base-pairing and thus prevent miRNAs from binding their target. For example, circTP63, a cell cycle related circRNA, is up-regulated in lung squamous cell carcinoma (LUSC) tissues and its up-regulation is correlated with larger tumor size and higher TNM stage in LUSC patients. Mechanically, circTP63 competitively binds to miR-873-3p and prevents miR-873-3p to decrease the level of FOXM1, which up-regulates CENPA and CENPB, and finally facilitates cell cycle progression (40). In colorectal cancer (CRC), circHIPK3 acts as the sponge of miR-7, which is a well-known tumor suppressor, to promote colorectal cancer growth and metastasis (41). In breast cancer, circFBXW7 acts as the sponge of miR-197-3p, which induces c-Myc degradation by up-regulating FBXW7 expression, to inhibit the malignant progression of triple-negative breast cancer (42). In bladder cancer, circ-ACVR2A could directly interact with miR-626 and acts as a miRNA sponge to regulate EYA4 expression, thus inhibiting bladder cancer cell proliferation and metastasis (43).

#### CircRNAs as Protein Decoys

CircRNAs that harbor RNA binding protein (RBP) binding motifs may serve as sponges/decoys of the corresponding proteins and regulate their functions. For example, Abdelmohsen et al. identified circPABPN1 in human cervical carcinoma HeLa cells, which suppressed the translation of nuclear poly(A) binding protein 1 (PABPN1) mRNA through sequestering the RBP Hu-antigen R (HUR) (44). By binding prescadillo homologue 1 (PES1, an essential 60S pre-ribosomal assembly factor), circANRIL was found to impair pre-rRNA processing and ribosome biogenesis, which further led to nucleolar stress and p53 activation (45). YAP (yes-associated protein), a key component of Hippo pathway which plays crucial roles in tumorigenesis, can inhibit apoptosis and promote proliferation and metastasis of cancer cells. Wu et al. showed that circYAP could bind with YAP mRNA and translation-associated protein eIF4G and PABP to negatively regulate the expression of YAP. Moreover, the malignant phenotypes can be reversed by the ectopic expression of circYAP, which is similar to silencing endogenous Yap (46).

#### CircRNAs Enhancing Protein Functions

CircRNAs may also enhance the function of particular proteins through circRNA-protein interactions. Sun et al. demonstrated that circMYBL2, generated from the cell cycle check point gene MYLB2, could promote the proliferation of FLT3-ITD+ cells *in vitro* and *in vivo* through enhancing the translational efficiency of FLT3 kinase *via* increasing the binding of polypyrimidine tract-binding protein 1 (PTBP1) to FLT3 messenger RNA (47). In addition, circEIF3J and circPAIP2 were demonstrated to be able to positively regulate the expression their parental genes through enhancing the function of transcription factors (20). Both circEIF3J and circPAIP2 were EIciRNAs residing in the nucleus, they could promote RNA polymerase II (Poll II)-mediated transcription by interacting with the U1 small nuclear ribonuleoprotein (snRNP)

#### CircRNAs as Protein Scaffolds or Recruitment

Some circRNAs have been shown to facilitate the colocalization of enzymes and their substrates through acting as protein scaffolds. Circ-Foxo3 physically binds to mouse double-minute 2 (MDM2) and mutant p53 through acting as protein scaffolds (48). Circ-Foxo3 was further demonstrated to facilitate MDM2-mdiated ubiquitylation of mutant p53, leading to proteasome-mediated degradation. CircRNAs may also recruit specific proteins to certain loci or subcellular compartments. For instance, Chen et al. found that circ-FECR1 could induce demethylation of CpG sites and promote transcription of FLI1 through recruiting TET1 to the promoter region of FLI1 (36).

#### CircRNAs Translating Into Peptides

Some circRNAs harbor internal ribosome entry site elements and AUG sites, which enables circRNAs to be translated to unique peptides under certain circumstances. Zhao et al. suggested that circE7, generated by oncogenic human papillomaviruses (HPVs), can be translated to produce E7 oncoprotein which is biologically functional and linked to the transforming properties of some HPV (49). Zhang et al. suggested that an 87-animo-acid peptide, encoded by circular form of the long intergenic non-protein-encoding RNA p53-induced transcript (LINC-PINT), directly interact with polymerase associated factor complex (PAF1c) and inhibited the transcriptional elongation of multiple oncogenes to suppresses glioblastoma cell proliferation *in vitro* and *in vivo* (50). Liang et al. showed that a novel 370-amino acid β-catenin isoform encoded by circRNA circβ-catenin could stabilize full-length β-catenin by antagonizing GSK3β-induced β-catenin phosphorylation and degradation, leading to activation of Wnt pathway, thus promoting liver cancer cell growth (51).

#### Interactions Between circRNAs and m6A Modification

N6-methyladenosine (m6A), which has been discovered in the early 1970s, and whose predominant accumulations around stop codons and 3’ untranslated regions (3’ UTRs) of mRNA with a typical consensus sequence RRACH (R = G or A and H = A, C, or U) have been reported (52–57), is one of the most common RNA modifications. Accumulating studies show that m6A play crucial roles in many different aspects including circadian rhythm, gene expression, cell differentiation, stress response, tumorigenesis, development, and inflammatory response (54, 58–63). Recently, Zhou et al. defined thousands of m6A circRNAs that showed cell-type-specific expression patterns (64). These circRNAs interact with m6A reader proteins YTHDF1 and YTHDF2, and m6A writer protein METTL3. Besides, Chen et al. also presented that the m6A reader YTHDC1 increase the cytoplasmic export of circRNA NOP2/Sun RNA methyltransferase 2 (circNSUN2), forming a circNSUN2-IGF2BP2-HMGA2 RNA-protein ternary complex in the cytoplasm contributing the stabilization of HMGA2 mRNA and the enhancement of colorectal liver metastasis (65). In addition, m6A writer protein METTL3 was reported to impact m6A modification of circZNF609, and the m6A reader proteins YTHDF1 was reported to regulate the backsplicing of circZNF609, suggesting the role of m6A in the biogenesis of circZNF609 (66). Besides, the translation of circRNAs was affected by m6A methylation (64), and the m6A modification on circRNAs can be recognized by mammalian cells to inhibit innate immunity by abrogating immune gene activation (67). All these studies expand our knowledge on the complex interactions between m6A modification and circRNAs.

In conclusion, circRNAs are prevalently expressed in human cancers and can exert its regulatory roles by acting as sponges/decoys for miRNAs and proteins, enhancing protein functions, protein scaffolds, recruiting proteins, or protein translation templates.

## Tumor Immunology and Immunotherapy

### Immune System in Human Cancers

The first indication of the immune system involvement in cancer was discovering the links between inflammation and cancer in 1863 (68). Endeavors have focused on how the immune system can be able to recognize and ultimately destroy cancer, which is made up of tumor and “self” cells. Cancer cells can express two types of tumor antigens: tumor-specific antigen (TSAs) and tumor-associated antigens (TAAs). TSAs are highly tumor-specific and are expressed only in tumor cells, while TAAs are more widely expressed in both tumor and normal cells (69). The immune system can respond to cancer cells in two ways, i.e. against TSAs or against TAAs. In immunosurveillance hypothesis, immune system recognizes malignant tumor cells as foreign agents and eliminates them (70). However, in the past decades, scientists found that cancer could actively deploy various tactics, which collectively termed “immune evasion mechanisms” and continuously develop diversity and complexity in late-stage, to delay, alter, or even stop anti-tumor immunity.

Interference on tumor immunology to intensify the immune response to eliminate tumor cells has provide novel insights on tumor therapy, i.e. immunotherapy, which includes immune checkpoint blockade (ICB) therapy, CAR T cell adoptive therapy, cancer vaccines, and oncolytic virus therapy.

### Immune Checkpoint Blockade Therapy

Tumor cells can overexpress some specific molecules such as PD-L1 and CTLA-4 to silence the immune response and these molecules are collectively termed as “immune checkpoints.” Programmed cell death (PD) pathway is the first and most characterized “immune checkpoints.” PD-1, a co-inhibitory receptor, is highly expressed on activated T cells, B lymphocytes, natural killer cells, and MDSCs. PD-1 expression can be induced by TCR-antigen engagement and common γ-chain cytokines like interleukin (IL)-2, IL-7, IL-15, and IL-21 in the effector phase of the immune response (71). PD-L1 and PD-L2 are two known ligands of PD-1 and their expression on healthy tissues are relatively low (72). Effector T cell exhausted and could be triggered apoptosis upon engaging with PD-L1 (73). Multiple cancers including melanoma, non-small cell lung cancer (NSCLC), breast cancer, and squamous cell head and neck cancer has been documented with up-regulated expression of PD-L1 (74–76). Therapeutic monoclonal antibodies directly against PD-1 and PD-L1, with avelumab for PD-L1 and nivolumab for PD-1, respectively, have been proven effective for treating multiple solid tumors. CTLA-4, mainly expressed on T cells, acts as a negative regulatory receptor of T cells. Upon the TCR engaging with antigens, the expression of CTLA-4 rapidly up-regulates. CTLA-4 can compete with CD28, a key co-stimulatory receptor on T cells, for the binding of the same ligands, CD80 and CD86. And CTLA-4 has a higher affinity than CD28 for both ligands, resulting in interference with the immune synapse and T-cell inactivation. Therapeutic anti-CTLA-4 monoclonal antibodies, such as ipilimumab, have achieved promising clinical outcomes in advanced melanoma. Although ICB are considered as a revolution of cancer treatment, many patients including microsatellite stable colorectal cancer (CRC), ovarian cancer, prostate cancer, and pancreatic ductal adenocarcinoma (PDA) rarely exhibit objective responses to ICB (77). There must be other mechanisms leveraged by tumor cells to suppress the immune response.

### Chimeric Antigen Receptor T Cell Adoptive Therapy

Chimeric antigen receptor T (CAR T) technique was first reported to transduce T cells with chimeric genes encoding single-chain antibodies that are linked to a transmembrane region and an intracellular domain encoding the signaling adaptor for the T cell receptor (78). CAR T cells could recognize tumor antigens independent of MHC presentation. By adopting CAR T technology, CAR T therapeutics genetically modified autologous T cells isolated from patients to express the CAR construct. After expansion, patient-derived genetically modified T cells were returned to patients to kill malignant cells. CAR T immunotherapy was demonstrated to redirect T cell killing to cells that express the antibody’s cognate antigen (79–81). Currently, the major targets of CAR T therapy is CD19, the B cell costimulatory receptor widely expressing on B cell leukemias and lymphomas (80, 82), showing highly therapeutic effects even in otherwise refractory diseases and can induce durable remissions (79, 83). Despite of impressive clinical outcomes in treating tumor patients, especially for those with lymphoma, CAR T therapy still have many problems, particularly for solid tumors (84). As CAR T therapy is based on direct recognition of tumor cells expressing CD19, deletion of the CAR-binding epitope frequently induces disease relapse (85). Beyond CD19, next-generation CAR T therapy will include novel targets such as CD22, which is a B cell regulatory receptor expressed by many B cell malignancies (83, 86). CD70 is also considered as a novel CAR T therapy target (87, 88).

### Cancer Vaccines

Efforts of cancer vaccines have been made to promote cancer-specific immune response, which generate antitumor immunity, especially cytotoxic CD8+ T cells that are specific to tumor antigens. Administration of tumor antigens (e.g. overexpressed antigens, cancer-testis antigens, oncofetal antigens, and mutated antigens) with antigen-presenting cells (APC, e.g. DCs, B cells, and monocytes) shows therapeutic promise (83, 89–91). Current cancer vaccination includes four major types, i.e. peptide-based vaccines, APC-based vaccines, tumor-based vaccines, and virus-based vaccines. The most commonly used cancer vaccine is the MHC class I restricted peptide epitopes that are from shared TAAs aiming to activate rare specific CD8+ T cells, which has shown substantial therapeutic effects (92–94). Peptide vaccines with adjuvant formulation, such as cytokines and toll-like receptor (TLR) ligands, showed significant clinical benefits (94–96). Multiple peptides can be given at the same time (97, 98) and combinations of multi-peptide vaccines and chemotherapy also indicated benefits (99). Among various types of APCs (e.g. peripheral blood mononuclear cells, activated B cells and dendritic cells), the heterogeneous populations of dendritic cells could efficiently process and present antigens to CD4+ and CD8+ T cells. Application of dendritic cells vaccines offered clinical efficiency (100–102). Tumor cells from killed mice could be used to immunize other mice by expressing immune stimulatory cytokines (e.g. GM-CSF) (103, 104). These findings offered the possibility of tumor cell- based immunotherapy. Tests using allogeneic cell lines or autologous tumor cells exhibited capability to activate immunity killing tumor cells (105, 106). Although huge endeavors have been made in cancer vaccinations, the effects of cancer vaccines are limited due to the difficulty in target antigens selection and immunosuppression from tumor microenvironment. Improvements in antigen choice and vaccine design will obtain better clinical outcomes.

### Oncolytic Virus Therapy

In the context of presenting tumor antigens, pathogens involvement can largely increase immune stimulation of tumor patients. Oncolytic viruses selectively replicate in tumor cells and kill them without harming normal cells, which can be genetically engineered or naturally occur (107). The most widely known virus vaccines for cancer are the human papillomavirus vaccines that are designed to prevent human papillomavirus (HPV) mediated cervical cancers. The oncolytic viruses have been used for decades, including adenoviruses, vaccinia viruses, and herpesviruses (91). Adenoviruses drive the transactivator early genes E1a and E1b and viral replication from tumor specific promoters. Taking advantage of natural infectivity, adenoviruses have been used to directly immunize with tumor antigens, where they are injected into muscle tissue as vectors (108, 109). The therapeutic efficacy of vaccinia viruses can be improved by genetically engineering with chemokine genes or combinations with costimulation (110). Oncolytic virus therapy has shown promising therapeutic values, but needs further exploration.

In conclusion, tumor immunology advances our understanding of the development of malignant tumor cells and their interactions with host, and immunotherapy has achieved great efforts in some malignant cancers. But tumor are a highly heterogeneous disease and tumor micro-environment are also complicated in different cancers. In the future, we should get more insights into complicated interplay between immune cells and tumor microenvironment, which will develop strategies to break the tumor evasion from immunosurveillance.

## CircRNAs and Tumor Immune Microenvironment

### Tumor Immune Microenvironment

Studies over several years has demonstrated that tumor cells could evade immune surveillance by establishing an immune-privileged microenvironment, which is functionally analogous to that of certain normal tissue (111, 112). Tumor immune microenvironment (TIME) is composed of various cell types except cancer cells, including tumor-associated macrophages, myeloid-derived suppressor cells (MDSCs), dendritic cells, cancer-associated fibroblasts cells, NK cells, tumor-associated neutrophils, and tumor-infiltrating lymphocytes. Over the past years scientists has realized that tumor cells could turn these cells to favor their progression and contribute to the immune escape. Firstly, accumulating evidences suggested that the vessels of TIME could control the extravasation of effector T cells from the circulatory system into tumors. Upon activated by the antigen present cells, the effector T cells traffic to the tumor *via* the circulatory system. For example, the apoptosis inducer Fas ligand (FAS-L) is highly expressed in the tumor vasculature of multiple tumor types including ovarian, colon, prostate, breast, bladder, and renal cancer, which substantially reduces the number of CD8+T cell infiltration into tumors. Accordingly, inhibition of Fas-L in preclinical models resulted in a significant increase of effector T cells in tumors and led to T cell dependent tumor suppression (113). Secondly, dendritic cells and MDSCs within the TIME can inhibit the immune response within the tumor *via* multiple mechanisms. After extravasation of cancer-specific T cells into tumor, it must locally replicate to further increase their numbers to kill the tumor cells effectively and also overcome barriers that restrict their distribution and the hostile elements of the TIME. MDSCs, key components of TIME, are broadly defined as myeloid cells and are different from mature myeloid cells (i.e. macrophages, DCs, neutrophils), which are terminally differentiated. MDSCs are consisted of myeloid progenitors, immature mononuclear cells (M-MDSCs), which are morphologically and phenotypically similar to monocytes, and immature polymorphonuclear (PMN-MDSCs), which are morphologically and phenotypically similar to neutrophils. M-MDSCs and PMN-MDSCs utilize different mechanisms to inhibit tumor immune response. M-MDSCs primarily utilize mechanisms associated with production of NO and cytokines to suppress both antigen-specific and nonspecific T cell response and have stronger suppressive activity than PMN-MDSCs (114). Other studies indicated that MDSCs and tumor-associated macrophage cells (TAMs) could also produce arginase-1, inducible nitric oxide synthase (iNOS) and reactive oxygen species (ROS) to suppress the proliferation of T cells within the tumor. Arginase-1 produced by MDSCs and TAMs within the TIME converts L-arginine, essential for the proliferation of T cells, to urea and L-ornithine, exhausting the pool of L-arginine within the TIME and thus impairing the proliferation of T cells. And MDSC-derived iNOS converts L-arginine to citrulline and NO, which suppresses T cell function by inhibiting JAK/STAT signaling, reducing MHC class II expression and inducing T cell apoptosis. While ROS and NO produced by MDSCs and TAMs result in nitration of the T cell receptor, which impairs the recognition of peptide antigens presented by MHC. Additionally, MDSCs can directly inhibit T cell response in a contact-dependent manner *via* membrane-bound TGF-β (115, 116). Furthermore, cancer associated-fibroblasts (CAFs) can prevent the effector T cells from accumulating in the vicinity of cancer cells within the TIME. Cancer-associated fibroblasts can leverage two methods to mediate this restriction. For the one hand, CAFs can produce extracellular matrix to exclude effector T cell. And studies found increased T cells movement out of the stromal regions and into contact with cancer cells when collagenase was added to reduce matrix rigidity. CAFs can also produce CXCL12 and IL-6 to exclude the effector T cells. Accordingly, administering an inhibitor of CXCR4, the receptor for CXCL12, to the PDA-bearing mice led to the rapid accumulation of effector T cells within the tumor and blockage IL-6 could improve T-cell trafficking, migration and tumor immunosuppression (117).

### The Relation Between circRNAs and Tumor Immune Microenvironment

Emerging studies showed that circRNAs played an important role in key components of tumor immune microenvironment (89, 118). Here we briefly review the interactions between key components of tumor immune microenvironment and circRNAs. One study suggested that circSLC8A1, derived from the SLC8A1 gene, could act as a sponge of miR-494, which is crucial for migration of MDSCs into tumor site and regulation of the production of ARG1 and iNOS, thus enhancing the tumor immune response. This evidence indicated that circRNAs could serve as potential targets by modulating the MDSC-mediated immune response. Natural killer cells (NK cells) play an important role in tumor immune surveillance, which possesses the ability to direct against tumor and infected cells without stimulation like B or T lymphocytes. They simultaneously express activating and inhibitory receptors that encounter target cells by the subtle balance of transmitted signals for activation or inhibition. NK cells primarily leveraged two tactics to against tumors. One is that NK cell can express apoptotic ligands such as TNF-related apoptosis-inducing ligand (TRAIL) and tumor necrosis factor (TNF) family members Fas-L and then interact with their related receptor on tumor cells which inducing the apoptosis of tumor cells. The other is that NK cell secrete perforin and granzyme to direct lyse tumor cells. In addition to direct against tumors, NK cells can exert “helper role” to against tumor cells. It was reported that NK cells could contribute to the accumulation of T-bet^+^ CD4^+^ T cells in the tumor site, promote the production of TNF-αand IFN-γ by tumor infiltrating CD8+T cells, suppress the expression of exhaustion marker PD-1 on these CD8+ T cells and promote the induction of tumor-specific T cell memory in the mouse model. Dysfunction of NK cells has been documented over the several years in TIME (119–122). And some studies suggested that circRNAs can enhance or attenuate the function of NK cells. Ma et al. showed that overexpression of circARSP91 (circRNA of AR-suppressed PABPC1 91bp) could enhance cytotoxicity of NK cells to HCC cells *via* up-regulating UL16 binding protein 1 (ULBP1) expression in HCC cells at the mRNA and protein levels (123). Interestingly, in the another study, Zhang et al. demonstrated that HCCs could secrete circUHRF1 in exosomal manner to inhibit NK cells-derived IFN-γ and TNF-α secretion by up-regulating the expression of TIM-3 *via* degradation of miR-449c-5p. Additionally, circUHRF1 could mediate the resistance to anti-PD1 immunotherapy in HCC patients (124). Macrophages, key components of innate immunity, mainly derived from embryonically and seeded in tissues, serve as the first-line defense against pathogens and antigen-presenting cells for cellular immunity (125). Macrophages can be divided into M1 and M2 macrophages based their catabolism of L-arginine. The M1 macrophages has a function of eliciting inflammation, while the M2 macrophages show anti-inflammatory effect. This concept may explain the heterogeneity of macrophages. M2 phenotype macrophages, primarily derived from circulating bone marrow monocytes, is induced by soluble factors secreted by cancer stem cells (CSCs) (126, 127). Studies over human tumor samples have indicated that a high number of macrophages, especially M2 macrophages, is closely related to worse clinical prognosis in numerous malignant cancers (128). Accumulating evidence suggested that tumor-associated macrophages could inhibit cytotoxic T cell response by the following mechanisms: depletion of essential metabolite required for T cell proliferation, inhibition of T cell functions *via* producing anti-inflammatory cytokines and activation of T cell checkpoint blockade *via* inhibitory receptors. One study indicated that the expression levels of circRNA-003780, circRNA-010056, and circRNA-010231 were high in M1 cells, while the expression levels of circRNA-003424, circRNA-013630, circRNA-001489, and circRNA-018127 were five times than those of M1 macrophage. Different expression levels of specific circRNAs in different polarization state of macrophages suggested that circRNAs may play an important role in the polarization of macrophages (129). Additionally, another study demonstrated that high expression level of circ-CDR1as was correlated with higher ratio of M2 macrophage, suggesting circ-CDR1as may be involved in the polarization of macrophages in TIME (130). Tumor infiltrating lymphocytes, considered as selected population of T-cell with a higher specific immunological reactivity against tumor cells than the non-infiltrating lymphocytes, are mainly composed of CD3^+^CD4^+^T cells and CD3^+^CD8^+^T cells. Multiple studies have showed that higher proportion of tumor infiltrating lymphocytes (TILs) predicted a better prognostics (131). It was reported that HCC patients with higher percentage of TILs displayed better clinical outcomes, suggesting the prognostic value of TILs for HCC patients. In addition, Weng et al., performed global circRNA microarray between plasma of HCC patients with high TILs and low TILs. The results suggested HCC patients with high TILs had lower expression of hsa_circ_0064428 and was negatively correlated with overall survival, tumor size and metastasis. It can be concluded that hsa_circ_0064428 functioned as a novel immune-associated prognostic biomarker for HCC patients (132).

In conclusion, reprogrammed TIME provides a “shield” for tumor cell and contributes immune therapy resistance. CircRNAs play an important role in tumor progression. Aberrant functions in the TIME caused by circRNAs can be valuable new targets to treat cancer or become novel biomarkers for immunotherapy.

## CircRNAs and Immunotherapy

**Figure 2:**
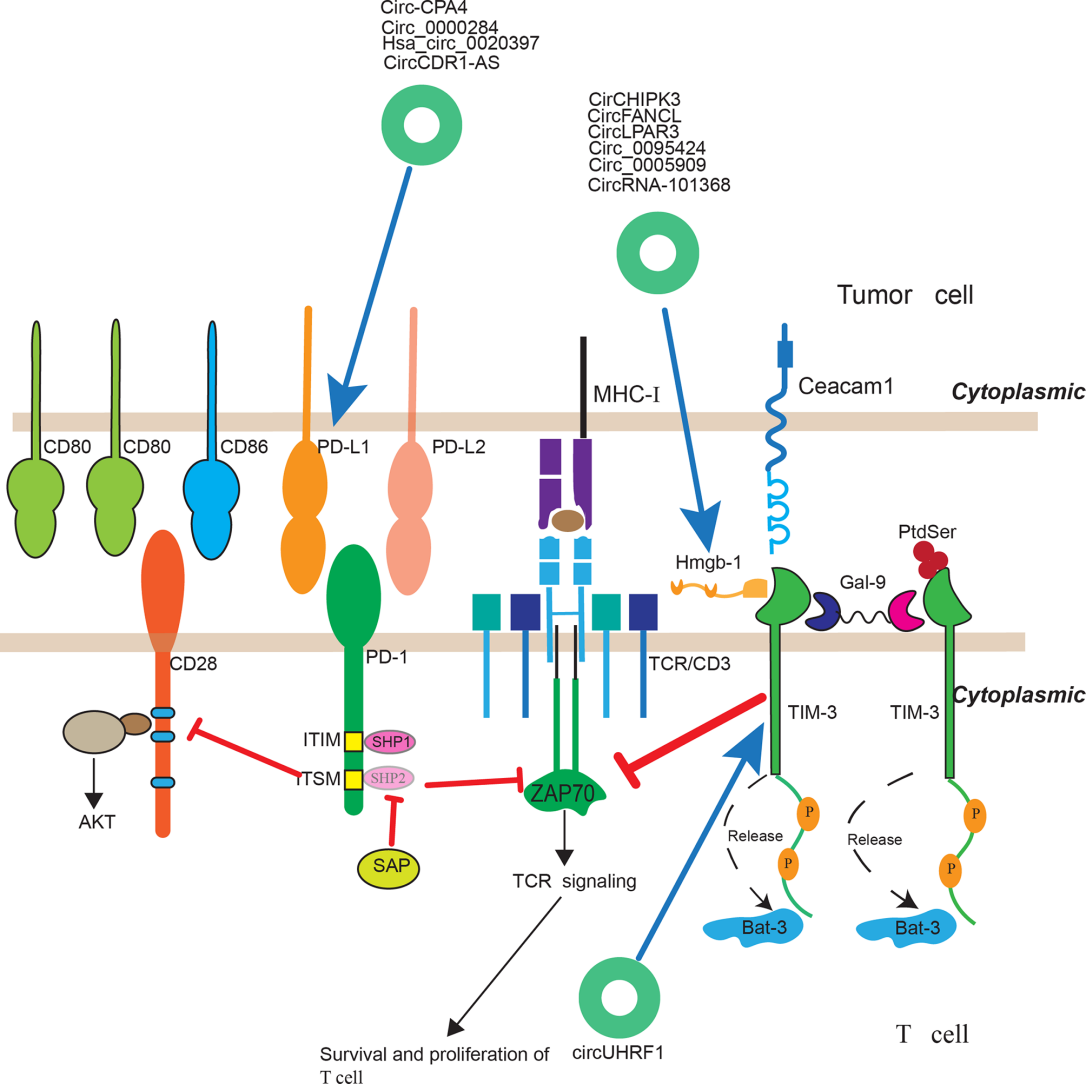
The regulatory roles of circRNAs in immunotherapy. CircRNAs have been implicated to play a regulatory role of immune checkpoints and have the potential to serve as a predictive biomarker of immune checkpoint therapy.

Cancer immunotherapy have achieved therapeutic advances in recent years and was named as 2013’s breakthrough of the year by *Science*. It highlights the importance of human immune system in treating cancer. Immune checkpoint inhibitors, using therapeutic antibodies including anti-CTLA4 and anti-PD1/L1 mAb, to unleash cytotoxic T cells in tumor microenvironment, has achieved great success in clinical practices. Accumulating studies suggested that B7-H1/PD-1 interaction was the major ways used by tumor cells to suppress immune response in both preclinical and clinical settings (133, 134). Anti-PD1 therapy have achieved higher objective response rates in patients and with much fewer immune-related adverse events (irAEs), which is the most characterized feature of this approach. It is effective in more than 25 different types of solid tumor and has favorable response-to-toxicity profile, with a 40% objective tumor response rate and a 7–12% grade 3–5 irAEs immune across multiple tumor types (135). Although anti-PD1 therapy has achieved great clinical success in most solid cancers, a considerable portion of patients did not benefit from anti-PD1 therapy. The reason is that the tumor microenvironment of different cancers of different patients are heterogeneous (136). For a successful immune-mediated elimination, it requires substantial leukocytes to infiltrate into tumor tissue and recognize the malignant cells. However, there exists significant difference across numerous tumors. For example, it was reported that effector T cells infiltrated in melanoma and breast cancer, but rarely infiltrated in pancreatic ductal adenocarcinoma. So pancreatic ductal adenocarcinoma did not response to anti-PD1 therapy. Clinically, cancer patients are broadly classified into three categories based on the level of tumor infiltrating leukocytes and B7-H1 expression level: (1) Types I and IV: There is lack of significant TILs in the TIME. (2) Types II: There exist many TILs in the TME and these TILs were over-regulated due to the effect of the B7-H1/PD-1 pathway. (3) Type III: there are many TILs in the TIME which are dysfunctional due to suppression by other molecular pathways (non-B7-H1/PD-1). Only patients classified as Types II can benefit from the anti-PD1 therapy (135). Thus understanding the dominant immune defects in TIME of patients is critical for cancer immunotherapy. And immune defects induced by tumor cells are highly heterogeneous (136). With the rapid development of cancer immunotherapy, there are many novel immune checkpoints including LAG3 (CD233), TIM, TIGIT (T cell immunoglobulin and ITIM domain), VISTA, B7-H3, BTLA, and siglec-15 emerging (137). And inhibitors targeting these checkpoints are on clinical trials. Interestingly, a study indicated that when the mice treated with anti-PD-1 mAb, the level of LAG-3 and CTLA-4 increased and treated with anti-LAG-3 mAb, the level of PD-1 were up-regulated. This suggested that blockade of a single immune checkpoint targets may led to the compensatory up-regulation of other checkpoint receptors in TME (138). This compensatory mechanism of immune checkpoints may be another mechanism of anti-PD1 therapy resistance in patients and also indicated that there existed common compensatory mechanism across different types of cancers. CircRNAs, as novel non-coding RNAs in the past few years, have been implicated in multiple physiological and pathophysiological conditions. And they can also play a regulatory role of immune checkpoints and have the potential to serve as a predictive biomarker of immune checkpoint therapy (**Figure 2**). One study suggested that hsa_circ_0020397 can regulate CRC cell viability, apoptosis, and invasion by promoting the expression of miR-138 target genes, telomerase reverse transcriptase (TERT), and programmed death-ligand 1 (PD-L1) (139). CircCDR1-AS is representative circular RNA that is associated with poor prognosis in gastrointestinal cancers including colon, liver, and pancreatic cancers. Tanaka et al. demonstrated that CircCDR1-AS can significantly increase the expression of PD-L1 at the surface of colon cancer cells *via* CMTM4 and CMTM6 and led to the poor prognosis of CRC cancer patients (140). In another study, Hong et al. indicated that circ-CPA4 could promote cell growth, mobility and epithelial-mesenchymal transition and inhibited cell deaths of NSCLC cells by up-regulating expression levels of PD-L1 *via* acting as an RNA sponge for let-7 miRNA. Moreover, circ-CPA4 could positively regulate exosomal PD-L1 derived from NSCLC cells, which promoted cell stemness and inactivated CD8^+^ T cells (141). Additionally, Li et al. found that circ_0000284 could up-regulate the expression of PD-L1 *via* binding miR-377-3p and thus promoting the progression of NSCLC (142). TIM-3, also called hepatitis A virus cellular receptor 2 (HAVCR2), is another intriguing immune checkpoint. The gene TIM, located on human chromosome 5q33.2, expresses a protein of 302 amino acid which belongs to Ig superfamily (IgSF). It was expressed on different immune cells including B cells, T cells, NK cells, DCs, Tregs, monocytes, and macrophages. And higher expression of TIM-3 was closely associated with poor prognosis in solid malignant. More importantly, accumulating evidence have verified that therapeutic benefit of TIM-3 blockade and inhibit tumor growth especially combined with anti-PD therapy. For the time being, there are at least eight anti-TIM-3 mAbs has been registered on *clinicaltrials.gov*. Anti-TIM-3 therapy combined with TSR-042 are already on phase II clinical trial for liver cancer (137). Zhang et al. reported circUHRF1, circular ubiquitin-like with PHD and ring finger domain 1 RNA (circUHRF1), is highly expressed in human HCC tissues and closely related to poor clinical prognosis of HCC patients. Mechanically, hepatocellular carcinoma (HCC) can secreted circUHRF1 in an exosomal manner, inhibiting the secretion of NK cell-derived IFN-γ and TNF-α and inhibiting NK cell function by up-regulating the expression of TIM-3 through degradation of miR-449c-5p (124).

One challenge for cancer immunotherapy is that tumor-induced immune defects not only occur among different patients but also extend to different areas in a single tumor lesion. It is important to evaluate immune response at the TIME level which require sequential tumor tissue collection and analyses. CircRNAs, presented as a stable covalently closed single RNA and can also be secreted in exosomal manner, can play a regulatory role in some of these immune checkpoints and have the potential to serve as a biomarker of immune checkpoint therapy response. Hence, more regulatory roles of circRNAs playing in these immune checkpoints need further investigation.

## Conclusions and Future Perspective

Due to the rapid progress of high-throughput sequencing and bioinformatics methodologies, researchers have unveiled the biogenesis and biological characteristics of circRNAs. Accumulating evidences exhibited that circRNAs are closely linked to immune cells in the tumor immune environment and are potentially to modulate or mark the infiltrating abundance of immune cells. Through regulating immune checkpoint genes, circRNAs are potentially capable to mediate therapeutic efficacy of immune checkpoint blockade therapy. The regulatory roles of circRNAs in cancer biology and therapy, especially tumor immunology and immunotherapy, need to be further explored. Several FDA-approved anti-cancer drugs were reported to be able target non-coding oncogenic RNAs. For example, miR-21 could be inhibited by compounds and its function could be ablated upon overexpressing pre-miR21. This finding indicates that onco-ncRNAs are druggable. However, there are no drugs specifically targeting onco-ncRNAs been clinically approved by FDA. Accumulating endeavors have been made to find small molecules targeting onco-ncRNAs. In terms of circRNA treatment, although no clinical trials have been reported, siRNAs targeting their unique conjunction cite, which can avoid targeting host gene, could be used to down-regulate specific onco-circRNAs to treat cancers. The major problem would be how to construct an efficient vector to deliver these siRNAs to tumor. Thus, ncRNAs treatment, we believe, will come true with the development of vector deliver system in the near future. To our best knowledge, the major possible negative effect of circRNA treatment could be mis-targeting. And this problem could be addressed by carefully designed siRNAs targeting the conjunction sites of circRNAs. In speaking of translational study of circRNAs, one study suggested that artificially designed circRNA could act as a miRNA sponge to sequester miR-122, which plays an important role in the transfer of hepatitis, to inhibit the replication of HCV (143). And another study showed that artificially designed circRNA sponging miR-132 and miR-212 could attenuate pressure overloaded-induced cardiac hypertrophy (144). These studies show a great potential for translational application of circRNAs. As we all known, there are many miRNAs associated with human diseases has been unraveled in the past years and one major functions of circRNA is to act as miRNA sponge to sequester miRNAs. In the future, not only circRNAs could be “druggable,” but also it could be “drug.” CircRNAs are characterized by their covalently closed loop structures and conjunction cites. When a siRNA specifically targeting conjunction cites of onco-circRNAs are used to treat cancer, it wouldn’t target mRNAs of host genes and this could be more precise than traditional ones. In addition, not only circRNAs could be targeted by siRNAs, but also artificially designed circRNAs could be delivered into cells to sponge onco-miRNAs to treat cancers.

Utilization of cutting-edge biotechnologies, such as single cell sequencing, the regulatory roles of circRNAs in tumor progression and immunology will be clearer. Thus, circRNAs will be employed in modulating the abundance or activity of immune cells and immune checkpoints expression, which will enhance the clinical efficiency of tumor immunotherapy.

## Author Contributions

ZF, SL, and CJ collected the materials. ZF and SL draw the figures and wrote the manuscript. All authors contributed to the article and approved the submitted version.

## Funding

This study is supported by the Shanghai General Hospital Startup Funding (02.06.01.20.06).

## Conflict of Interest

The authors declare that the research was conducted in the absence of any commercial or financial relationships that could be construed as a potential conflict of interest.

## Abbreviations

CircRNAs, circular RNAs; EciRNAs, exonic circRNAs; EIciRNAs, exon-intron circRNAs; CiRNAs, intronic RNAs; f-circRNAs, fusion circRNAs; rt-circRNAs, read-through circRNAs; TIME, tumor immune microenvironment; RNA-seq, RNA sequencing; pre-mRNA, pre-messenger RNA; LUSC, lung squamous cell carcinoma; CRC, colorectal cancer; RBP, RNA binding protein; PABPN1, poly(A) binding protein 1; HUR, Hu-antigen R; PES1, prescadillo homologue 1; YAP, yes associated protein; PTBP1, polypyrimidine tract-binding protein 1; MDM2, mouse double-minute 2; HPV, human papillomaviruses; PAF1c, polymerase associated factor complex; TSA, tumor-specific antigen; TSA, tumor-specific antigen; TAA, tumor-associated antigens; PD, programmed cell death; IL, interleukin; NSCLC, non-small cell lung cancer; PDA, pancreatic ductal adenocarcinoma; ICB, immune checkpoint blockade; snRNP, small nuclear ribonuleoprotein; Pol II, polymerase II.

## References

[1] Nahand JS Jamshidi S Hamblin MR Mahjoubin-Tehran M Vosough M Jamali M Circular RNAs: New Epigenetic Signatures in Viral Infections. Front Microbiol (2020) 11:1853. 10.3389/fmicb.2020.01853 32849445 PMC7412987

[2] Yousefi F Shabaninejad Z Vakili S Derakhshan M Movahedpour A Dabiri H TGF-β and WNT signaling pathways in cardiac fibrosis: Non-coding RNAs come into focus. Cell Commun Signal (2020) 18:1–16. 10.1186/s12964-020-00555-4 32517807 PMC7281690

[3] Abbaszadeh-Goudarzi K Radbakhsh S Pourhanifeh MH Khanbabaei H Davoodvandi A Fathizadeh H Circular RNA and Diabetes: Epigenetic Regulator with Diagnostic Role. Curr Mol Med (2020) 20:516–26. 10.2174/1566524020666200129142106 31995005

[4] Vo JN Cieslik M Zhang Y Shukla S Xiao L Zhang Y The Landscape of Circular RNA in Cancer. Cell (2019) 176:869–81. 10.1016/j.cell.2018.12.021 PMC6601354 30735636

[5] Starke S Jost I Hung L Bindereif A Exon Circularization Requires Canonical Splice Signals. CellReports (2015) 10:103–11. 10.1016/j.celrep.2014.12.002 25543144

[6] Park OH Ha H Lee Y Boo SH Kwon DH Song HK Endoribonucleolytic Cleavage of m6A-Containing RNAs by RNase P/MRP Complex. Mol Cell (2019) 74:494–507.e8. 10.1016/j.molcel.2019.02.034 30930054

[7] Hansen TB Wiklund ED Bramsen JB Villadsen SB Statham AL Clark SJ MiRNA-dependent gene silencing involving Ago2-mediated cleavage of a circular antisense RNA. EMBO J (2011) 30:4414–22. 10.1038/emboj.2011.359 PMC3230379 21964070

[8] Cocquerelle C Mascrez B Hetuin D Bailleul B Mis-splicing yields circular RNA molecules. FASEB J Vol (2016) 7:155–60. 10.1096/fasebj.7.1.7678559 7678559

[9] Pasman Z Been DM Garcia-Blanco AM Exon circularization in mammalian nuclear extracts. RNA (1996) 2:603–10. PMC1369399 8718689

[10] Cocquerelle C Daubersies P Majerus MA Kerckaert JP Bailleul B Splicing with inverted order of exons occurs proximal to large introns. EMBO J (1992) 11:1095–8. 10.1002/j.1460-2075.1992.tb05148.x PMC556550 1339341

[11] Nigro JM Cho KR Fearon ER Kern SE Ruppert JM Oliner JD Scrambled exons. Cell (1991) 64:607–13. 10.1016/0092-8674(91)90244-S 1991322

[12] Wang PL Bao Y Yee MC Barrett SP Hogan GJ Olsen MN Circular RNA is expressed across the eukaryotic tree of life. PloS One (2014) 9:e90859. 10.1371/journal.pone.0090859 24609083 PMC3946582

[13] Ivanov A Memczak S Wyler E Torti F Porath HT Orejuela MR Analysis of intron sequences reveals hallmarks of circular RNA biogenesis in animals. Cell Rep (2015) 10:170–7. 10.1016/j.celrep.2014.12.019 25558066

[14] Jeck WR Sorrentino JA Wang K Slevin MK Burd CE Liu J Circular RNAs are abundant, conserved, and associated with ALU repeats. RNA (2013) 19:426. 10.1261/rna.035667.112 PMC3543092 23249747

[15] Salzman J Chen RE Olsen MN Wang PL Brown PO Cell-Type Specific Features of Circular RNA Expression. PloS Genet (2013) 9:e1003777. 10.1371/annotation/f782282b-eefa-4c8d-985c-b1484e845855 24039610 PMC3764148

[16] Westholm JO Miura P Olson S Shenker S Joseph B Sanfilippo P Genome-wide Analysis of Drosophila Circular RNAs Reveals Their Structural and Sequence Properties and Age-Dependent Neural Accumulation. Cell Rep (2014) 9:1966–80. 10.1016/j.celrep.2014.10.062 PMC4279448 25544350

[17] Kristensen LS Andersen MS Stagsted LVW Ebbesen KK Hansen TB Kjems J The biogenesis, biology and characterization of circular RNAs. Nat Rev Genet (2019) 20:675–91. 10.1038/s41576-019-0158-7 31395983

[18] Ma S Kong S Wang F Ju S CircRNAs : biogenesis, functions, and role in drug-resistant Tumours. Mol Cancer (2020) 19:119. 10.1186/s12943-020-01231-4 PMC7409473 32758239

[19] Guo JU Agarwal V Guo H Bartel DP Expanded identification and characterization of mammalian circular RNAs. Genome Biol (2014) 15:1–14. 10.1186/s13059-014-0409-z PMC4165365 25070500

[20] Li Z Huang C Bao C Chen L Lin M Wang X Exon-intron circular RNAs regulate transcription in the nucleus. Nat Struct Mol Biol (2015) 22:256–64. 10.1038/nsmb.2959 25664725

[21] Zhang Y Zhang XO Chen T Xiang JF Yin QF Xing YH Circular Intronic Long Noncoding RNAs. Mol Cell (2013) 51:792–806. 10.1016/j.molcel.2013.08.017 24035497

[22] Jeong JC Lo-Coco F Guarnerio J Bezzi M Naldini MM Berry K Oncogenic Role of Fusion-circRNAs Derived from Cancer-Associated Chromosomal Translocations. Cell (2016) 165:289–302. 10.1016/j.cell.2016.03.020 27040497

[23] Ashwal-Fluss R Meyer M Pamudurti NR Ivanov A Bartok O Hanan M CircRNA Biogenesis competes with Pre-mRNA splicing. Mol Cell (2014) 56:55–66. 10.1016/j.molcel.2014.08.019 25242144

[24] Liang D Tatomer DC Luo Z Wu H Yang L Chen LL The Output of Protein-Coding Genes Shifts to Circular RNAs When the Pre-mRNA Processing Machinery Is Limiting. Mol Cell (2017) 68:940–54. 10.1016/j.molcel.2017.10.034 PMC5728686 29174924

[25] Zhang XO Wang HB Zhang Y Lu X Chen LL Yang L Complementary sequence-mediated exon circularization. Cell (2014) 159:134–47. 10.1016/j.cell.2014.09.001 25242744

[26] Conn SJ Pillman KA Toubia J Conn VM Salmanidis M Phillips CA The RNA binding protein quaking regulates formation of circRNAs. Cell (2015) 160:1125–34. 10.1016/j.cell.2015.02.014 25768908

[27] Eger N Schoppe L Schuster S Laufs U Boeckel JN Circular RNA splicing. Adv Exp Med Biol (2018) 1087:41–52. 10.1007/978-981-13-1426-1_4 30259356

[28] Shukla S Kavak E Gregory M Imashimizu M Shutinoski B Kashlev M CTCF-promoted RNA polymerase II pausing links DNA methylation to splicing. Nature (2011) 479:74–9. 10.1038/nature10442 PMC7398428 21964334

[29] Rinaldi L Datta D Serrat J Morey L Solanas G Avgustinova A Dnmt3a and Dnmt3b Associate with Enhancers to Regulate Human Epidermal Stem Cell Homeostasis. Cell Stem Cell (2016) 19:491–501. 10.1016/j.stem.2016.06.020 27476967

[30] Li S Han L Circular RNAs as promising biomarkers in cancer : detection, function, and beyond. Genome Med (2019) 11:15. 10.1186/s13073-019-0629-7 PMC6427893 30894216

[31] Borran S Ahmadi G Rezaei S Anari MM Modabberi M Azarash Z Circular RNAs: New players in thyroid cancer. Pathol Res Pract (2020) 216:153217. 10.1016/j.prp.2020.153217 32987339

[32] Shabaninejad Z Vafadar A Movahedpour A Ghasemi Y Namdar A Fathizadeh H Circular RNAs in cancer: New insights into functions and implications in ovarian cancer. J Ovarian Res (2019) 12:1–12. 10.1186/s13048-019-0558-5 31481095 PMC6724287

[33] Naeli P Pourhanifeh MH Karimzadeh MR Shabaninejad Z Movahedpour A Tarrahimofrad H Circular RNAs and gastrointestinal cancers: Epigenetic regulators with a prognostic and therapeutic role. Crit Rev Oncol Hematol (2020) 145:102854. 10.1016/j.critrevonc.2019.102854 31877535 PMC6982584

[34] Hua JT Chen S He HH Landscape of Noncoding RNA in Prostate Cancer. Trends Genet (2019) 35:840–51. 10.1016/j.tig.2019.08.004 31623872

[35] Yang L Fu J Zhou Y Circular RNAs and Their Emerging Roles in Immune Regulation. Front Immunol (2018) 9:2977. 10.3389/fimmu.2018.02977 PMC6305292 30619334

[36] Chen N Zhao G Yan X Lv Z Yin H Zhang S A novel FLI1 exonic circular RNA promotes metastasis in breast cancer by coordinately regulating TET1 and DNMT1. Genome Biol (2018) 19:1–14. 10.1186/s13059-018-1594-y 30537986 PMC6290540

[37] Pamudurti NR Bartok O Jens M Ashwal-Fluss R Stottmeister C Ruhe L Translation of CircRNAs. Mol Cell (2017) 66:9–21. 10.1016/j.molcel.2017.02.021 28344080 PMC5387669

[38] Memczak S Jens M Elefsinioti A Torti F Krueger J Rybak A Circular RNAs are a large class of animal RNAs with regulatory potency. Nature (2013) 495:333–8. 10.1038/nature11928 23446348

[39] Du WW Yang W Liu E Yang Z Dhaliwal P Yang BB Foxo3 circular RNA retards cell cycle progression via forming ternary complexes with p21 and CDK2. Nucleic Acids Res (2016) 44:2846–58. 10.1093/nar/gkw027 PMC4824104 26861625

[40] Cheng Z Yu C Cui S Wang H Jin H Wang C circTP63 functions as a ceRNA to promote lung squamous cell carcinoma progression by upregulating FOXM1. Nat Commun (2019) 10:3200. 10.1038/s41467-019-11162-4 31324812 PMC6642174

[41] Zeng K Chen X Xu M Liu X Hu X Xu T CircHIPK3 promotes colorectal cancer growth and metastasis by sponging miR-7 article. Cell Death Dis (2018) 9:417. 10.1038/s41419-018-0454-8 29549306 PMC5856798

[42] Ye F Gao G Zou Y Zheng S Zhang L Ou X circFBXW7 Inhibits Malignant Progression by Sponging miR-197-3p and Encoding a 185-aa Protein in Triple-Negative Breast Cancer. Mol Ther - Nucleic Acids (2019) 18:88–98. 10.1016/j.omtn.2019.07.023 31536884 PMC6796723

[43] Dong W Bi J Liu H Yan D He Q Zhou Q Circular RNA ACVR2A suppresses bladder cancer cells proliferation and metastasis through miR-626/EYA4 axis. Mol Cancer (2019) 18:1–16. 10.1186/s12943-019-1025-z 31101108 PMC6524247

[44] Abdelmohsen K Panda AC Munk R Grammatikakis I Dudekula DB De S Identification of HuR target circular RNAs uncovers suppression of PABPN1 translation by CircPABPN1. RNA Biol (2017) 14:361–9. 10.1080/15476286.2017.1279788 PMC5367248 28080204

[45] Holdt LM Stahringer A Sass K Pichler G Kulak NA Wilfert W Circular non-coding RNA ANRIL modulates ribosomal RNA maturation and atherosclerosis in humans. Nat Commun (2016) 7:12429. 10.1038/ncomms12429 27539542 PMC4992165

[46] Wu N Yuan Z Du KY Fang L Lyu J Zhang C Translation of yes-associated protein (YAP) was antagonized by its circular RNA via suppressing the assembly of the translation initiation machinery. Cell Death Differ (2019) 26:2758–73. 10.1038/s41418-019-0337-2 PMC7224378 31092884

[47] Sun YM Wang WT Zeng ZC Chen TQ Han C Pan Q CircMYBL2, a circRNA from MYBL2, regulates FLT3 translation by recruiting PTBP1 to promote FLT3-ITD AML progression. Blood (2019) 134:1533–46. 10.1182/blood.2019000802 PMC6839953 31387917

[48] Du WW Fang L Yang W Wu N Awan FM Yang Z Induction of tumor apoptosis through a circular RNA enhancing Foxo3 activity. Cell Death Differ (2017) 24:357–70. 10.1038/cdd.2016.133 PMC5299715 27886165

[49] Zhao J Lee EE Kim J Yang R Chamseddin B Ni C Transforming activity of an oncoprotein-encoding circular RNA from human papillomavirus. Nat Commun (2019) 10:2300 31127091 10.1038/s41467-019-10246-5 PMC6534539

[50] Zhang M Zhao K Xu X Yang Y Yan S Wei P A peptide encoded by circular form of LINC-PINT suppresses oncogenic transcriptional elongation in glioblastoma. Nat Commun (2018) 9:4475. 10.1038/s41467-018-06862-2 30367041 PMC6203777

[51] Liang W Wong C Liang P Shi M Cao Y Rao S Translation of the circular RNA circβ-catenin promotes liver cancer cell growth through activation of the Wnt pathway. Genome Biol (2019) 20:84. 10.1186/s13059-019-1685-4 31027518 PMC6486691

[52] Dominissini D Moshitch-Moshkovitz S Schwartz S Salmon-Divon M Ungar L Osenberg S Topology of the human and mouse m6A RNA methylomes revealed by m6A-seq. Nature (2012) 485:201–6. 10.1038/nature11112 22575960

[53] Meyer KD Saletore Y Zumbo P Elemento O Mason CE Jaffrey SR Comprehensive analysis of mRNA methylation reveals enrichment in 3′ UTRs and near stop codons. Cell (2012) 149:1635–46. 10.1016/j.cell.2012.05.003 PMC3383396 22608085

[54] Zhao BS Roundtree IA He C Post-transcriptional gene regulation by mRNA modifications. Nat Rev Mol Cell Biol (2016) 18:31–42. 10.1038/nrm.2016.132 27808276 PMC5167638

[55] Alarcón CR Lee H Goodarzi H Halberg N Tavazoie SF N6-methyladenosine marks primary microRNAs for processing. Nature (2015) 519:482–5. 10.1038/nature14281 PMC4475635 25799998

[56] Lee M Kim B Kim VN Emerging roles of RNA modification: M6A and U-Tail. Cell (2014) 158:980–7. 10.1016/j.cell.2014.08.005 25171402

[57] Desrosiers R Friderici K Rottman F Identification of methylated nucleosides in messenger RNA from Novikoff hepatoma cells. Proc Natl Acad Sci USA (1974) 71:3971–5. 10.1073/pnas.71.10.3971 PMC434308 4372599

[58] Edens BM Vissers C Su J Arumugam S Xu Z Shi H FMRP Modulates Neural Differentiation through m6A-Dependent mRNA Nuclear Export. Cell Rep (2019) 28:845–54.e5. 10.1016/j.celrep.2019.06.072 PMC6687293 31340148

[59] Engel M Eggert C Kaplick PM Eder M Röh S Tietze L The Role of m6A/m-RNA Methylation in Stress Response Regulation. Neuron (2018) 99:389–403.e9. 10.1016/j.neuron.2018.07.009 30048615 PMC6069762

[60] Yu R Li Q Feng Z Cai L Xu Q m6A reader YTHDF2 regulates lps-induced inflammatory response. Int J Mol Sci (2019) 20:1323. 10.3390/ijms20061323 PMC6470741 30875984

[61] Lan Q Liu PY Haase J Bell JL Huttelmaier S Liu T The critical role of RNA M6A methylation in cancer. Cancer Res (2019) 79:1285–92. 10.1158/0008-5472.CAN-18-2965 30894375

[62] Fustin JM Doi M Yamaguchi Y Hida H Nishimura S Yoshida M XRNA-methylation-dependent RNA processing controls the speed of the circadian clock. Cell (2013) 155:793. 10.1016/j.cell.2013.10.026 24209618

[63] Dai D Wang H Zhu L Jin H Wang X N6-methyladenosine links RNA metabolism to cancer progression. Cell Death Dis (2018) 9:124. 10.1038/s41419-017-0129-x PMC5833385 29374143

[64] Zhou C Molinie B Daneshvar K Pondick JV Wang J Van Wittenberghe N Genome-Wide Maps of m6A circRNAs Identify Widespread and Cell-Type-Specific Methylation Patterns that Are Distinct from mRNAs. Cell Rep (2017) 20:2262–76. 10.1016/j.celrep.2017.08.027 PMC5705222 28854373

[65] Chen RX Chen X Xia LP Zhang JX Pan ZZ Ma XD N 6-methyladenosine modification of circNSUN2 facilitates cytoplasmic export and stabilizes HMGA2 to promote colorectal liver metastasis. Nat Commun (2019) 10:1–15. 10.1038/s41467-019-12651-2 31619685 PMC6795808

[66] Di Timoteo G Dattilo D Centrón-Broco A Colantoni A Guarnacci M Rossi F Modulation of circRNA Metabolism by m6A Modification. Cell Rep (2020) 31:107641. 10.1016/j.celrep.2020.107641 32402287

[67] Yang Y Fan X Mao M Song X Wu P Zhang Y Extensive translation of circular RNAs driven by N6-methyladenosine. Cell Res (2017) 27:626–41. 10.1038/cr.2017.31 PMC5520850 28281539

[68] Galon J Bruni D Tumor Immunology and Tumor Evolution: Intertwined Histories. Immunity (2020) 52:55–81. 10.1016/j.immuni.2019.12.018 31940273

[69] Calabrese L Velcheti V Checkpoint immunotherapy: Good for cancer therapy, bad for rheumatic diseases. Ann Rheum Dis (2017) 76:1–3. 10.1136/annrheumdis-2016-209782 27566797

[70] Melief CJM Finn OJ Cancer immunology. N Engl J Med (2008) 358:2704–15. 10.1056/NEJMra072739 18565863

[71] Good-Jacobson KL Szumilas CG Chen L Sharpe AH Tomayko MM Shlomchik MJ PD-1 regulates germinal center B cell survival and the formation and affinity of long-lived plasma cells. Nat Immunol (2010) 11:535–42. 10.1038/ni.1877 PMC2874069 20453843

[72] Ghiotto M Gauthier L Serriari N Pastor S Truneh A Nunès JA PD-L1 and PD-L2 differ in their molecular mechanisms of interaction with PD-1. Int Immunol (2010) 22:651–60. 10.1093/intimm/dxq049 PMC3168865 20587542

[73] Latchman Y Wood CR Chernova T Chaudhary D Borde M Chernova I PD-L2 is a second ligand for PD-1 and inhibits T cell activation. Nat Immunol (2001) 2:261–8. 10.1038/85330 11224527

[74] Velcheti V Schalper KA Carvajal DE Anagnostou VK Syrigos KN Sznol M Programmed death ligand-1 expression in non-small cell lung cancer. Lab Invest (2014) 94:107–16. 10.1038/labinvest.2013.130 PMC6125250 24217091

[75] McLaughlin J Han G Schalper KA Carvajal-Hausdorf D Pelekanou V Rehman J Quantitative assessment of the heterogeneity of PD-L1 expression in non-small-cell lung cancer. JAMA Oncol (2016) 2:46–54. 10.1001/jamaoncol.2015.3638 26562159 PMC4941982

[76] Vassilakopoulou M Avgeris M Velcheti V Kotoula V Rampias T Chatzopoulos K Evaluation of PD-L1 Expression and Associated Tumor-Infiltrating Lymphocytes in Laryngeal Squamous Cell Carcinoma. Clin Cancer Res (2016) 22:704–13. 10.1158/1078-0432.CCR-15-1543 26408403

[77] Brahmer JR Tykodi SS Chow LQM Hwu WJ Topalian SL Hwu P Safety and activity of anti-PD-L1 antibody in patients with advanced cancer. N Engl J Med (2012) 366:2455–65. 10.1056/NEJMoa1200694 PMC3563263 22658128

[78] Yang Y Cancer immunotherapy : harnessing the immune system to battle cancer. J Clin Invest (2015) 125:3335–7. 10.1172/JCI83871 PMC4588312 26325031

[79] Park JH Rivière I Gonen M Wang X Sénéchal B Curran KJ Long-term follow-up of CD19 CAR therapy in acute lymphoblastic leukemia. N Engl J Med (2018) 378:449–59. 10.1056/NEJMoa1709919 PMC6637939 29385376

[80] Neelapu SS Locke FL Bartlett NL Lekakis LJ Miklos DB Jacobson CA Axicabtagene ciloleucel CAR T-cell therapy in refractory large B-Cell lymphoma. N Engl J Med (2017) 377:2531–44. 10.1056/NEJMoa1707447 PMC5882485 29226797

[81] Schuster SJ Svoboda J Chong EA Nasta SD Mato AR Anak Ö Chimeric antigen receptor T Cells in refractory B-Cell lymphomas. N Engl J Med (2017) 377:2545–54. 10.1056/NEJMoa1708566 PMC5788566 29226764

[82] Maude SL Laetsch TW Buechner J Rives S Boyer M Bittencourt H Tisagenlecleucel in children and young adults with B-cell lymphoblastic leukemia. N Engl J Med (2018) 378:439–48. 10.1056/NEJMoa1709866 PMC5996391 29385370

[83] Dougan M Dranoff G Dougan SK Cancer immunotherapy: Beyond checkpoint blockade. Annu Rev Cancer Biol (2019) 3:55–75. 10.1146/annurev-cancerbio-030518-055552 PMC10400018 37539076

[84] Xia A-L Wang X-C Lu Y-J Lu X-J Sun B Chimeric-antigen receptor T (CAR-T) cell therapy for solid tumors: challenges and opportunities. Oncotarget (2017) 8:90521–31. 10.18632/oncotarget.19361 PMC5685771 29163850

[85] Maude SL Frey N Shaw PA Aplenc R Barrett DM Bunin NJ Chimeric antigen receptor T cells for sustained remissions in leukemia. N Engl J Med (2014) 371:1507–17. 10.1056/NEJMoa1407222 PMC4267531 25317870

[86] Fry TJ Shah NN Orentas RJ Stetler-Stevenson M Yuan CM Ramakrishna S CD22-targeted CAR T cells induce remission in B-ALL that is naive or resistant to CD19-targeted CAR immunotherapy. Nat Med (2018) 24:20–8. 10.1038/nm.4441 PMC5774642 29155426

[87] Shaffer DR Savoldo B Yi Z Chow KKH Kakarla S Spencer DM T cells redirected against CD70 for the immunotherapy of CD70-positive malignancies. Blood (2011) 117:4304–14. 10.1182/blood-2010-04-278218 PMC3087480 21304103

[88] Jin L Ge H Long Y Yang C Chang YE Mu L CD70, a novel target of CAR T-cell therapy for gliomas. Neuro Oncol (2018) 20:55–65. 10.1093/neuonc/nox116 28651374 PMC5761579

[89] Xu Z Li P Fan L Wu M The potential role of circRNA in tumor immunity regulation and immunotherapy. Front Immunol (2018) 9:1–11. 10.3389/fimmu.2018.00009 29403493 PMC5786515

[90] Sahin U Türeci Ö Personalized vaccines for cancer immunotherapy. Sci (80- ) (2018) 359:1355–60. 10.1126/science.aar7112 29567706

[91] Butterfield LH Cancer vaccines. BMJ (2015) 350:1–14. 10.1136/bmj.h988 PMC4707521 25904595

[92] Zhao X Bose A Komita H Taylor JL Chi N Lowe DB Vaccines Targeting Tumor Blood Vessel Antigens Promote CD8 + T Cell-Dependent Tumor Eradication or Dormancy in HLA-A2 Transgenic Mice. J Immunol (2012) 188:1782–8. 10.4049/jimmunol.1101644 PMC3273624 22246626

[93] Komita H Zhao X Taylor JL Sparvero LJ Amoscato AA Alber S CD8+ T-cell responses against hemoglobin-β prevent solid tumor growth. Cancer Res (2008) 68:8076–84. 10.1158/0008-5472.CAN-08-0387 PMC2597529 18829566

[94] Kirkwood JM Lee S Moschos S Albertini MR Michalak JC Sander C Immunogenicity and antitumor effects of vaccination with peptide vaccine +/- granulocyte-monocyte colony-stimulating factor and/or IFIN-α2b in advanced metastatic melanoma: Eastern cooperative oncology group phase II trial E1696. Clin Cancer Res (2009) 15:1443–51. 10.1158/1078-0432.CCR-08-1231 PMC2759898 19228745

[95] Slingluff CL Lee S Zhao F Chianese-Bullock KA Olson WC Butterfield LH A randomized phase II trial of multiepitope vaccination with melanoma peptides for cytotoxic T cells and helper T cells for patients with metastatic melanoma (E1602). Clin Cancer Res (2013) 19:4228–38. 10.1158/1078-0432.CCR-13-0002 PMC3813832 23653149

[96] Pollack IF Jakacki RI Butterfield LH Hamilton RL Panigrahy A Potter DM Antigen-specific immune responses and clinical outcome after vaccination with glioma-associated antigen peptides and polyinosinic-polycytidylic acid stabilized by lysine and carboxymethylcellulose in children with newly diagnosed malignant brainstem and n. J Clin Oncol (2014) 32:2050–8. 10.1200/JCO.2013.54.0526 PMC4067943 24888813

[97] Slingluff CL Petroni GR Chianese-Bullock KA Smolkin ME Ross MI Haas NB Randomized multicenter trial of the effects of melanoma-associated helper peptides and cyclophosphamide on the immunogenicity of a multipeptide melanoma vaccine. J Clin Oncol (2011) 29:2924–32. 10.1200/JCO.2010.33.8053 PMC3138719 21690475

[98] Walter S Weinschenk T Stenzl A Zdrojowy R Pluzanska A Szczylik C Multipeptide immune response to cancer vaccine IMA901 after single-dose cyclophosphamide associates with longer patient survival. Nat Med (2012) 18:1254–61. 10.1038/nm.2883 22842478

[99] Disis ML Gad E Herendeen DR Phan-Lai V Park KH Cecil DL A multiantigen vaccine targeting neu, IGFBP-2, and IGF-IR prevents tumor progression in mice with preinvasive breast disease. Cancer Prev Res (2013) 6:1273–82. 10.1158/1940-6207.CAPR-13-0182 PMC3864759 24154719

[100] Giordano FJ Ping P Mckirnan MD Nozaki S Demaria AN Dillmann WH Vaccination of patients with B-cell lymphoma using autologous antigen-pulsed dendritic cells. Nat Med (1996) 2:534–9. 10.1038/nm0596-534 8564842

[101] Dummer R Burg G Schadendorf D Vaccination of melanoma patients with peptide- or tumor lysate-pulsed dendritic cells. Nat Med (1998) 4:328–32. 10.1038/nm0398-328 9500607

[102] Banchereau J Palucka AK Dhodapkar M Burkeholder S Taquet N Rolland A Immune and clinical responses in patients with metastatic melanoma to CD34+ progenitor-derived dendritic cell vaccine. Cancer Res (2001) 61:6451–8. 11522640

[103] Dranoff G Jaffee E Lazenby A Golumbek P Levitsky H Brose K Vaccination with irradiated tumor cells engineered to secrete murine granulocyte-macrophage colony-stimulating factor stimulates potent, specific, and long-lasting anti-tumor immunity. Proc Natl Acad Sci USA (1993) 90:3539–43. 10.1073/pnas.90.8.3539 PMC46336 8097319

[104] McBride WH Thacker JD Comora S Economou JS Kelley D Hogge D Genetic Modification of a Murine Fibrosarcoma to Produce Interleukin 7 Stimulates Host Cell Infiltration and Tumor Immunity. Cancer Res (1992) 52:3931–7. 1617669

[105] Le DT Brockstedt DG Nir-Paz R Hampl J Mathur S Nemunaitis J A live-attenuated listeria vaccine (ANZ-100) and a live-attenuated listeria vaccine expressing mesothelin (CRS-207) for advanced cancers: Phase I studies of safety and immune induction. Clin Cancer Res (2012) 18:858–68. 10.1158/1078-0432.CCR-11-2121 PMC3289408 22147941

[106] Kim TS Chopra A O-Sullivan IS Cohen EP Enhanced immunity to breast cancer in mice immunized with fibroblasts transfected with a complementary DNA expression library from breast cancer cells: Enrichment of the vaccine for immunotherapeutic cells. J Immunother (2006) 29:261–73. 10.1097/01.cji.0000197097.46100.bb 16699369

[107] Fukuhara H Ino Y Todo T Oncolytic virus therapy: A new era of cancer treatment at dawn. Cancer Sci (2016) 107:1373–9. 10.1111/cas.13027 PMC5084676 27486853

[108] Meng WS Butterfield LH Ribas A Dissette VB Heller JB Miranda GA α-Fetoprotein-specific tumor immunity induced by plasmid prime-adenovirus boost genetic vaccination. Cancer Res (2001) 61:8782–6. 11751399

[109] Butterfield LH Economou JS Gamblin TC Geller DA Alpha fetoprotein DNA prime and adenovirus boost immunization of two hepatocellular cancer patients. J Transl Med (2014) 12:1–9. 10.1186/1479-5876-12-86 24708667 PMC4021640

[110] John LB Howland LJ Flynn JK West AC Devaud C Duong CP Oncolytic virus and anti-4-1BB combination therapy elicits strong antitumor immunity against established cancer. Cancer Res (2012) 72:1651–60. 10.1158/0008-5472.CAN-11-2788 22315352

[111] Vinay DS Ryan EP Pawelec G Talib WH Stagg J Elkord E Immune evasion in cancer: Mechanistic basis and therapeutic strategies. Semin Cancer Biol (2015) 35:S185–98. 10.1016/j.semcancer.2015.03.004 25818339

[112] Cervantes-Villagrana RD Albores-García D Cervantes-Villagrana AR García-Acevez SJ Tumor-induced neurogenesis and immune evasion as targets of innovative anti-cancer therapies. Signal Transduct Target Ther (2020) 5:99. 10.1038/s41392-020-0205-z 32555170 PMC7303203

[113] Motz GT Santoro SP Wang LP Garrabrant T Lastra RR Hagemann IS Tumor endothelium FasL establishes a selective immune barrier promoting tolerance in tumors. Nat Med (2014) 20:607–15. 10.1038/nm.3541 PMC4060245 24793239

[114] Marvel D Gabrilovich DI Myeloid-derived suppressor cells in the tumor microenvironment: Expect the unexpected. J Clin Invest (2015) 125:3356–64. 10.1172/JCI80005 PMC4588239 26168215

[115] Brown JM Recht L Strober S The Promise of Targeting Macrophages in Cancer Therapy. Clin Cancer Res (2017) 23:3241–50. 10.1158/1078-0432.CCR-16-3122 PMC5529121 28341752

[116] Veglia F Perego M Gabrilovich D Myeloid-derived suppressor cells coming of age review-article. Nat Immunol (2018) 19:108–19. 10.1038/s41590-017-0022-x PMC5854158 29348500

[117] Feig C Jones JO Kraman M Wells RJB Deonarine A Chan DS Targeting CXCL12 from FAP-expressing carcinoma-associated fibroblasts synergizes with anti-PD-L1 immunotherapy in pancreatic cancer. Proc Natl Acad Sci USA (2013) 110:20212–7. 10.1073/pnas.1320318110 PMC3864274 24277834

[118] Song H Liu Q Liao Q Circular RNA and tumor microenvironment. Cancer Cell Int (2020) 20:1–15. 10.1186/s12935-020-01301-z 32518520 PMC7268656

[119] Sun C Sun HY Xiao WH Zhang C Tian ZG Natural killer cell dysfunction in hepatocellular carcinoma and NK cell-based immunotherapy. Acta Pharmacol Sin (2015) 36:1191–9. 10.1038/aps.2015.41 PMC4648180 26073325

[120] Cong J Wang X Zheng X Wang D Fu B Sun R Dysfunction of Natural Killer Cells by FBP1-Induced Inhibition of Glycolysis during Lung Cancer Progression. Cell Metab (2018) 28:243–55.e5. 10.1016/j.cmet.2018.06.021 30033198

[121] Merino A Miller JS Cichocki F Merino A Zhang B Dougherty P Chronic stimulation drives human NK cell dysfunction and epigenetic reprograming Find the latest version : Chronic stimulation drives human NK cell dysfunction and epigenetic reprograming. J Clin Invest (2019) 129:3770–85. 10.1172/JCI125916 PMC6715389 31211698

[122] Piątkiewicz P Miłek T Bernat-Karpińska M Ohams M Czech A Ciostek P The dysfunction of nk cells in patients with type 2 diabetes and colon cancer. Arch Immunol Ther Exp (Warsz) (2013) 61:245–53. 10.1007/s00005-013-0222-5 23456207

[123] Ma Y Zhang C Zhang B Yu H Yu Q circRNA of AR-suppressed PABPC1 91 bp enhances the cytotoxicity of natural killer cells against hepatocellular carcinoma via upregulating UL16 binding protein 1. Oncol Lett (2019) 17:388–97. 10.3892/ol.2018.9606 PMC6313186 30655779

[124] Zhang PF Zhang PF Zhang PF Gao C Gao C Huang XY Cancer cell-derived exosomal circUHRF1 induces natural killer cell exhaustion and may cause resistance to anti-PD1 therapy in hepatocellular carcinoma. Mol Cancer (2020) 19:1–15. 10.1186/s12943-020-01222-5 32593303 PMC7320583

[125] Pathria P Louis TL Varner JA Targeting Tumor-Associated Macrophages in Cancer. Trends Immunol (2019) 40:310–27. 10.1016/j.it.2019.02.003 30890304

[126] Qian BZ Li J Zhang H Kitamura T Zhang J Campion LR CCL2 recruits inflammatory monocytes to facilitate breast-tumour metastasis. Nature (2011) 475:222–5. 10.1038/nature10138 PMC3208506 21654748

[127] Komohara Y Fujiwara Y Ohnishi K Shiraishi D Takeya M Contribution of macrophage polarization to metabolic diseases. J Atheroscler Thromb (2016) 23:10–7. 10.5551/jat.32359 26412584

[128] Komohara Y Jinushi M Takeya M Clinical significance of macrophage heterogeneity in human malignant tumors. Cancer Sci (2014) 105:1–8. 10.1111/cas.12314 24168081 PMC4317877

[129] Zhang Y Zhang Y Li X Zhang M Lv K Microarray analysis of circular RNA expression patterns in polarized macrophages. Int J Mol Med (2017) 39:373–9. 10.3892/ijmm.2017.2852 PMC5358696 28075448

[130] Zou Y Zheng S Deng X Yang A Xie X Tang H The role of circular RNA CDR1as/cirs-7 in regulating tumor microenvironment: A pan-cancer analysis. Biomolecules (2019) 9:1–13. 10.3390/biom9090429 PMC6770779 31480381

[131] Badalamenti G Fanale D Incorvaia L Barraco N Listì A Maragliano R Role of tumor-infiltrating lymphocytes in patients with solid tumors: Can a drop dig a stone? Cell Immunol (2019) 343:103753. 10.1016/j.cellimm.2018.01.013 29395859

[132] Weng Q Chen M Li M Zheng YF Shao G Fan W Global microarray profiling identified hsa-circ-0064428 as a potential immune-associated prognosis biomarker for hepatocellular carcinoma. J Med Genet (2019) 56:32–8. 10.1136/jmedgenet-2018-105440 30120213

[133] Zou W Wolchok JD Chen L PD-L1 (B7-H1) and PD-1 pathway blockade for cancer therapy: Mechanisms, response biomarkers, and combinations. Sci Transl Med (2016) 8:328rv4. 10.1126/scitranslmed.aad7118 PMC4859220 26936508

[134] Ribas A Wolchok JD Cancer immunotherapy using checkpoint blockade. Sci (80- ) (2018) 359:1350–5. 10.1126/science.aar4060 PMC7391259 29567705

[135] Sanmamed MF Chen L A Paradigm Shift in Cancer Immunotherapy: From Enhancement to Normalization. Cell (2018) 175:313–26. 10.1016/j.cell.2018.09.035 PMC6538253 30290139

[136] Schalper KA Carvajal-Hausdorf D McLaughlin J Altan M Velcheti V Gaule P Differential expression and significance of PD-L1, IDO-1, and B7-H4 in human lung cancer. Clin Cancer Res (2017) 23:370–8. 10.1158/1078-0432.CCR-16-0150 PMC6350535 27440266

[137] Qin S Xu L Yi M Yu S Wu K Luo S Novel immune checkpoint targets: Moving beyond PD-1 and CTLA-4. Mol Cancer (2019) 18:1–14. 10.1186/s12943-019-1091-2 31690319 PMC6833286

[138] Huang RY Francois A McGray AR Miliotto A Odunsi K Compensatory upregulation of PD-1, LAG-3, and CTLA-4 limits the efficacy of single-agent checkpoint blockade in metastatic ovarian cancer. Oncoimmunology (2017) 6:e1249561. 10.1080/2162402X.2016.1249561 28197366 PMC5283642

[139] Zhang X Xu L Wang F Hsa_circ_0020397 regulates colorectal cancer cell viability, apoptosis, and invasion by promoting the expression of the miR-138 targets TERT and PD-L1. Cell Biol Int (2017) 41:1056–64. 10.1002/cbin.10826 28707774

[140] Tanaka E Miyakawa Y Kishikawa T Seimiya T Iwata T Funato K Expression of circular RNA CDR1-AS in colon cancer cells increases cell surface PD-L1 protein levels. Oncol Rep (2019) 42:1459–66. 10.3892/or.2019.7244 31322270

[141] Hong W Xue M Jiang J Zhang Y Gao X Circular RNA circ-CPA4/ let-7 miRNA/PD-L1 axis regulates cell growth, stemness, drug resistance and immune evasion in non-small cell lung cancer (NSCLC). J Exp Clin Cancer Res (2020) 39:1–19. 10.1186/s13046-020-01648-1 32746878 PMC7397626

[142] Li L Zhang Q Lian K Circular RNA circ_0000284 plays an oncogenic role in the progression of non-small cell lung cancer through the miR-377-3p-mediated PD-L1 promotion. Cancer Cell Int (2020) 20:1–11. 10.1186/s12935-020-01310-y 32550825 PMC7298744

[143] Jost I Shalamova LA Gerresheim GK Niepmann M Bindereif A Rossbach O Functional sequestration of microRNA-122 from Hepatitis C Virus by circular RNA sponges. RNA Biol (2018) 15:1032–9. 10.1080/15476286.2018.1435248 PMC6161685 29486652

[144] Lavenniah A Luu TDA Li YP Lim TB Jiang J Ackers-Johnson M Engineered Circular RNA Sponges Act as miRNA Inhibitors to Attenuate Pressure Overload-Induced Cardiac Hypertrophy. Mol Ther (2020) 28:1506–17. 10.1016/j.ymthe.2020.04.006 PMC7264434 32304667

